# Supplementary material for: Isoliquiritigenin Inhibits the Growth of Colorectal Cancer Cells through the ESR2/PI3K/AKT Signalling Pathway
Source: Pharmaceuticals (Basel). 2023 Dec 27;17(1):43. doi: 10.3390/ph17010043 (PMC10820227; doi:10.3390/ph17010043)
Supplement: Supplementary file 1 [file pharmaceuticals-17-00043-s001.zip › Figure S1. Fig 5A Supplementary-WB.pdf]

Figure S1. Fig.5A (Results: Regulation of ISL on ERβ/PI3K/Akt signal)

HCT116(Original images in Fig 5)

All experimental samples and controls were derived from the same experiment and were treated under the same western blotting conditions.

ESR2 69KD

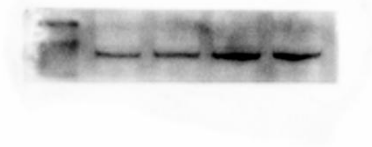

PIK3CG 121KD

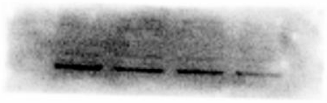

AKT

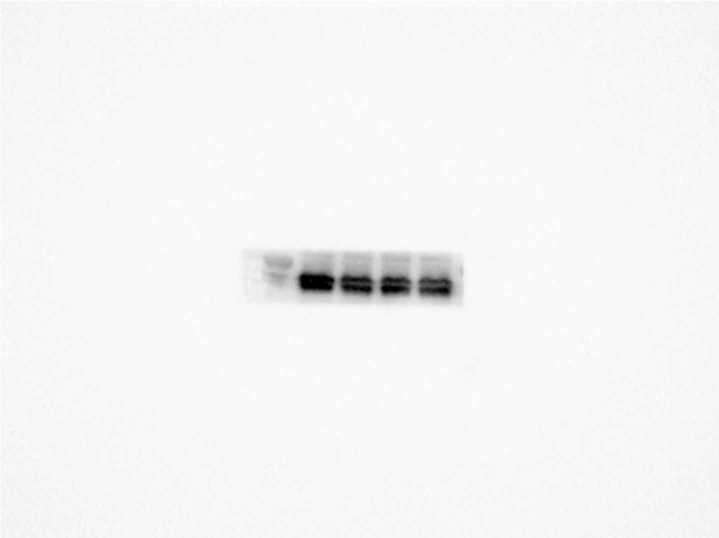

56KD

P-AKT

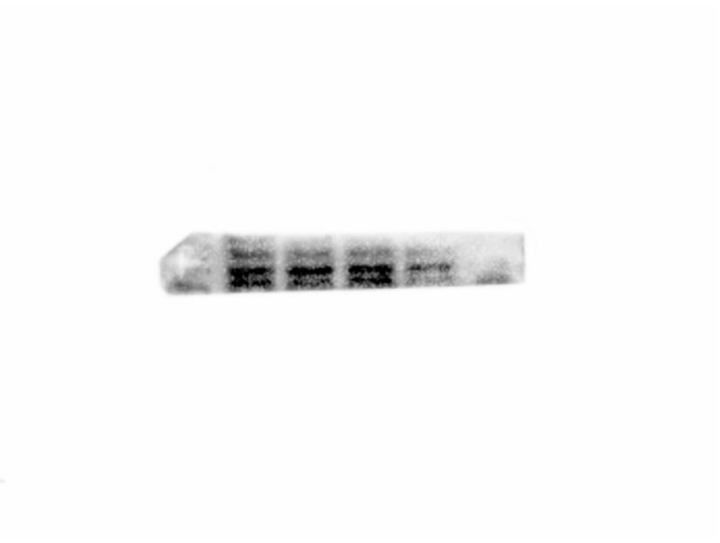

60KD

NF-κB

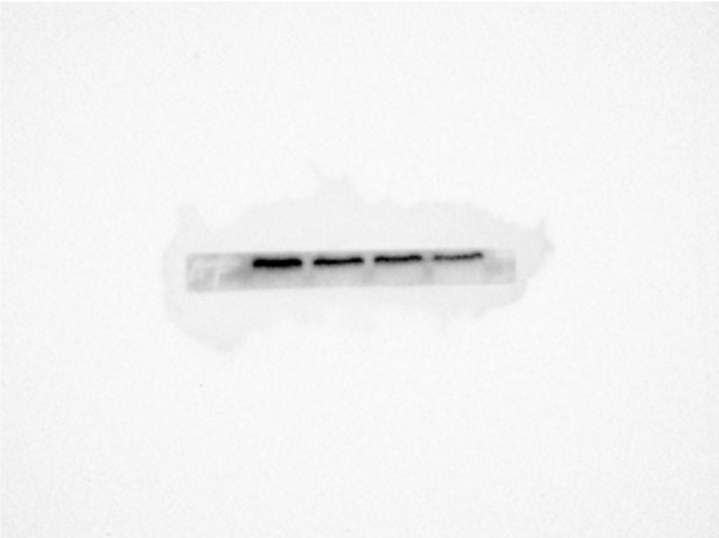

65KD

P-GSK3β

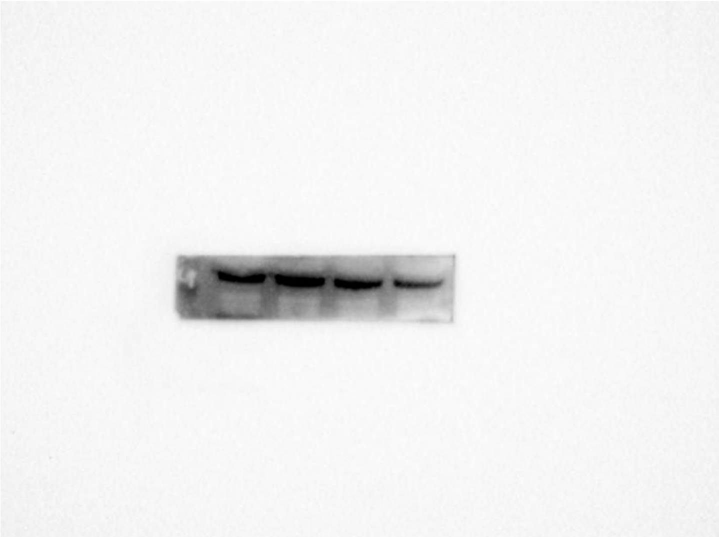

48KD

GSK3β

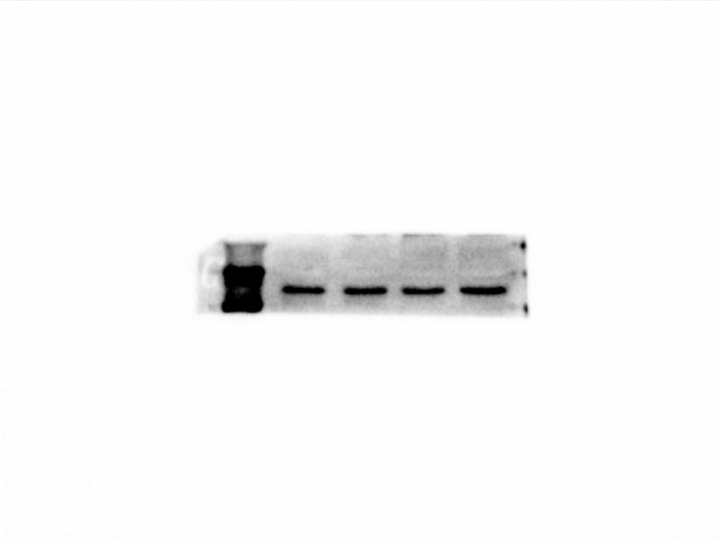

46KD

CDK1

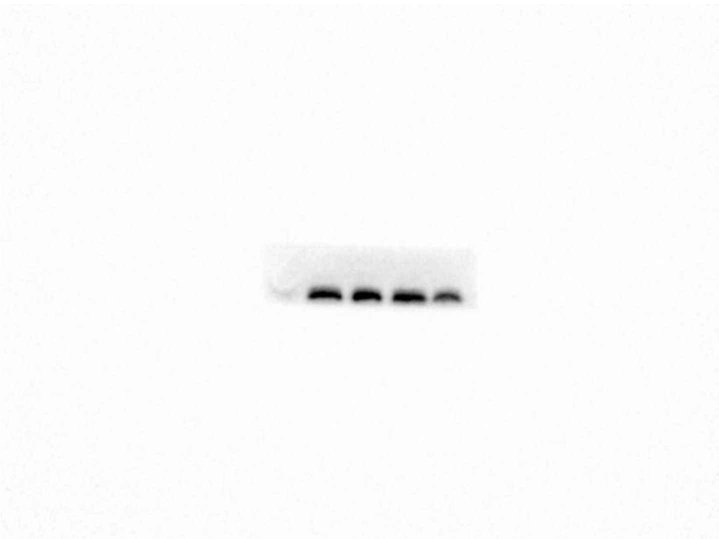

34KD

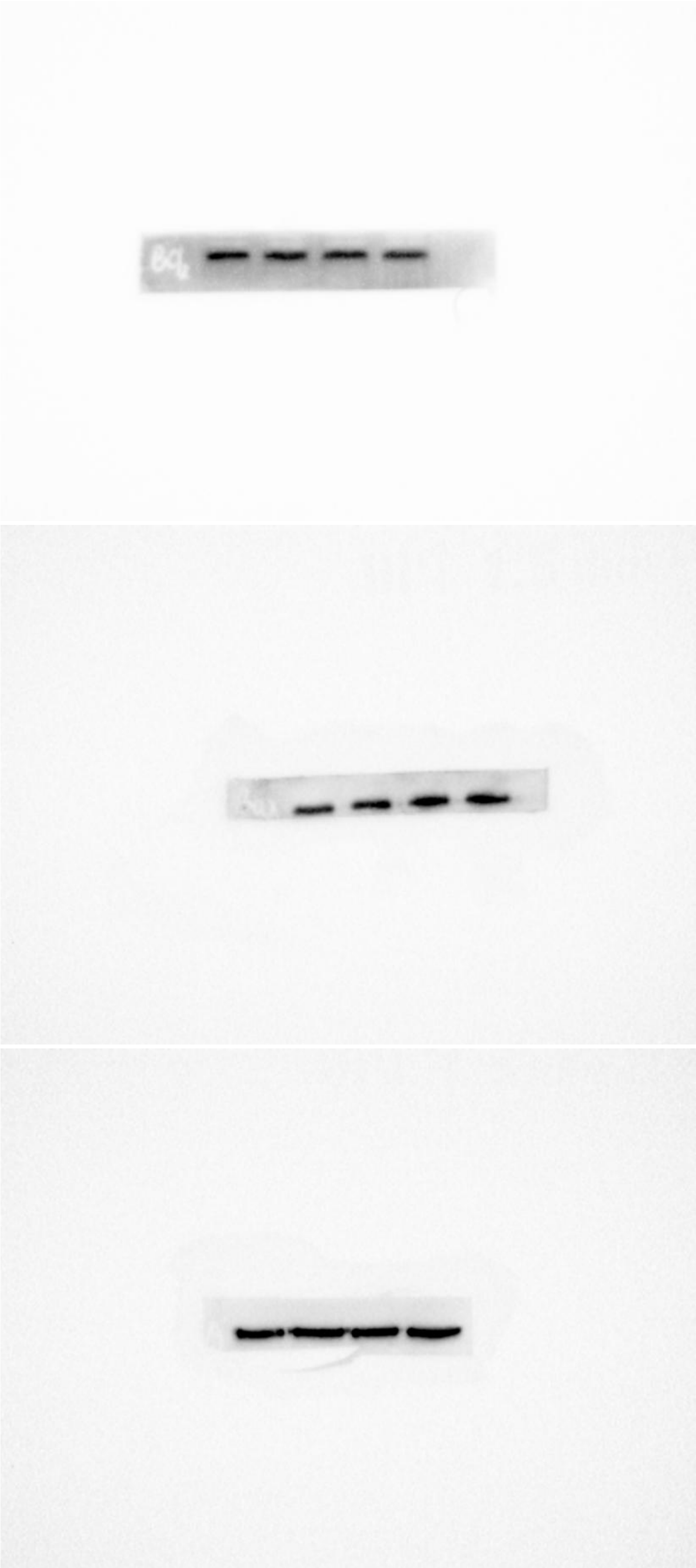

Bcl2 26KD

Bax 23KD

β-actin 42KD

Full-length blots of ESR2, PI3KCG, AKT, P-AKT, NK-κB, GSK3β, P-GSK3β, CDK1, Bcl-2, Bax and β-actin. From right to left: 0 μM, 20 μM, 40 μM, 80 μM. The samples derive from the same experiment.

HCT116(Repeated experiments)

All experimental samples and controls were derived from the same experiment and were treated under the same western blotting conditions.

ESR2

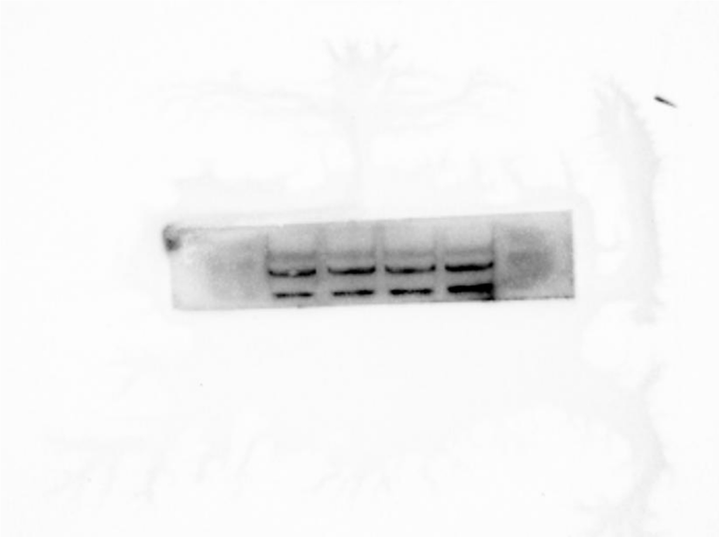

69KD

PIK3CG

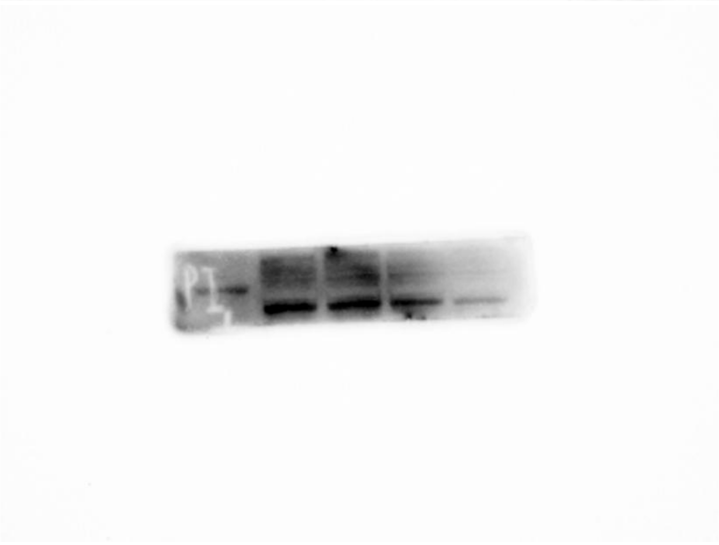

121KD

AKT

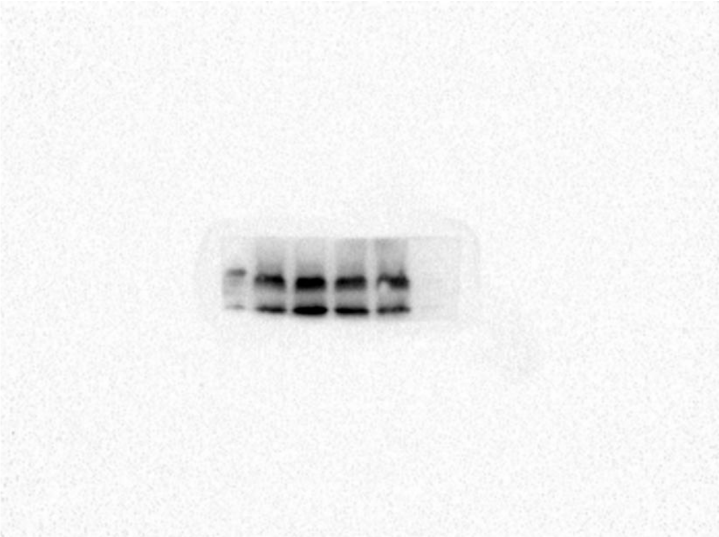

56KD

P-AKT

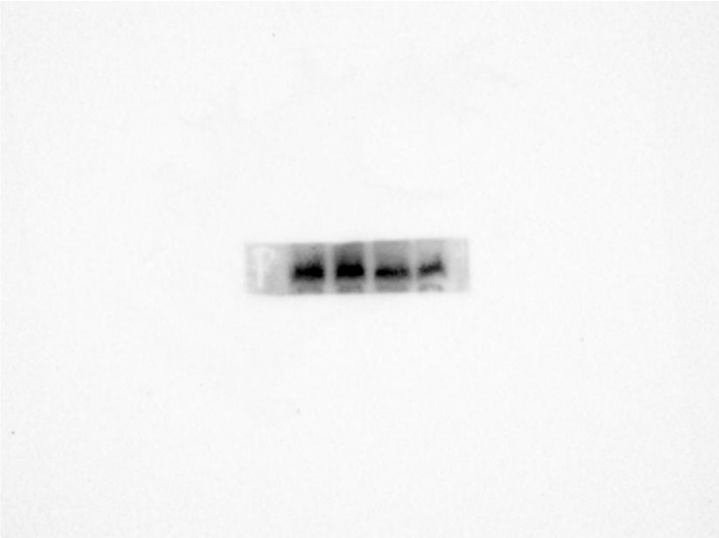

60KD

NF-κB

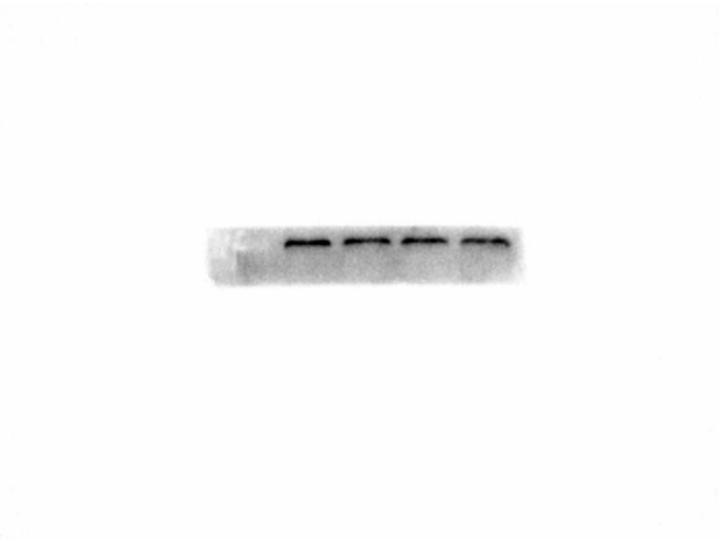

65KD

GSK3β

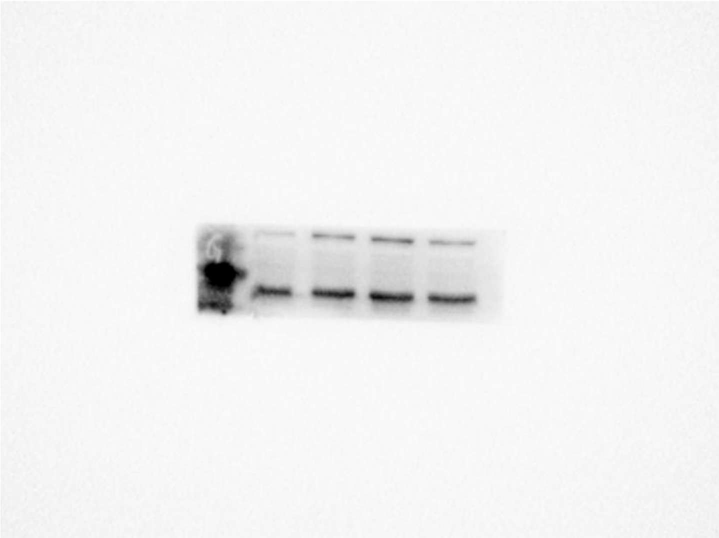

46KD

P-GSK3β

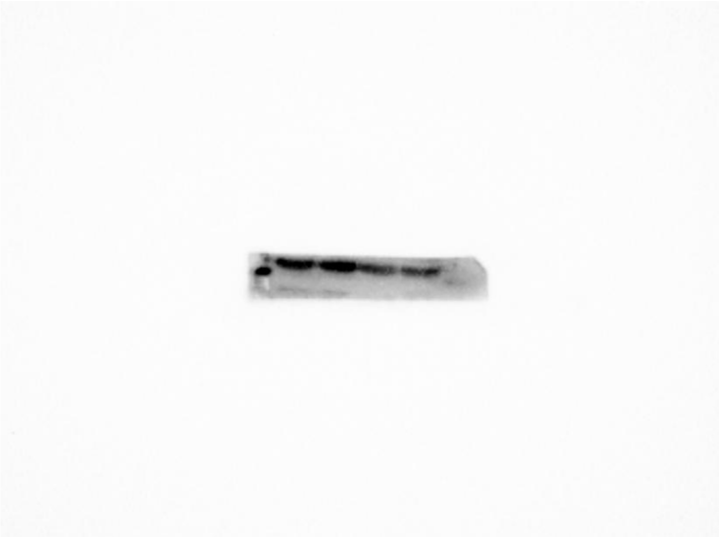

48KD

CDK1

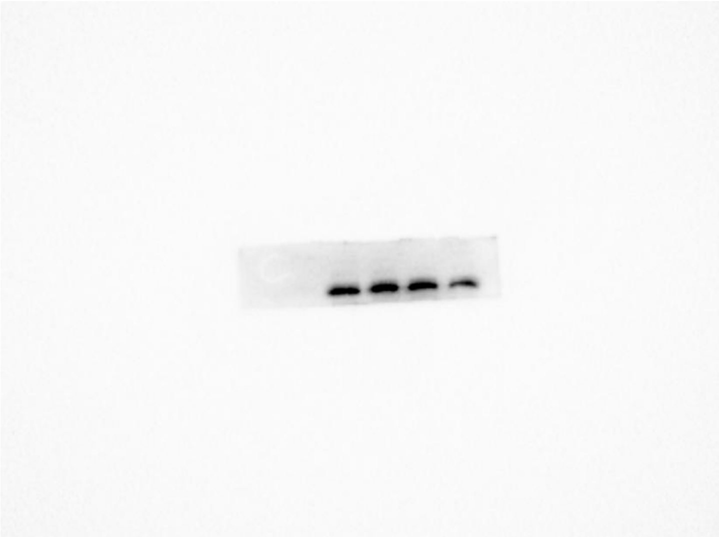

34KD

Bcl2

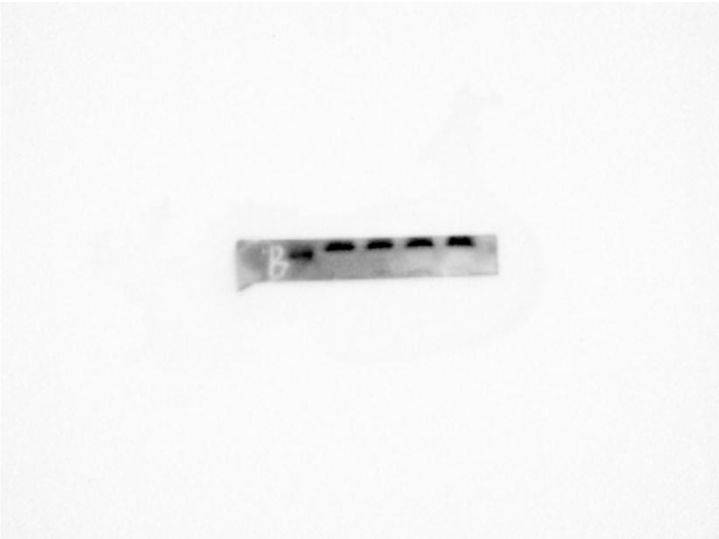

26KD

Bax 23KD

β-actin 42KD

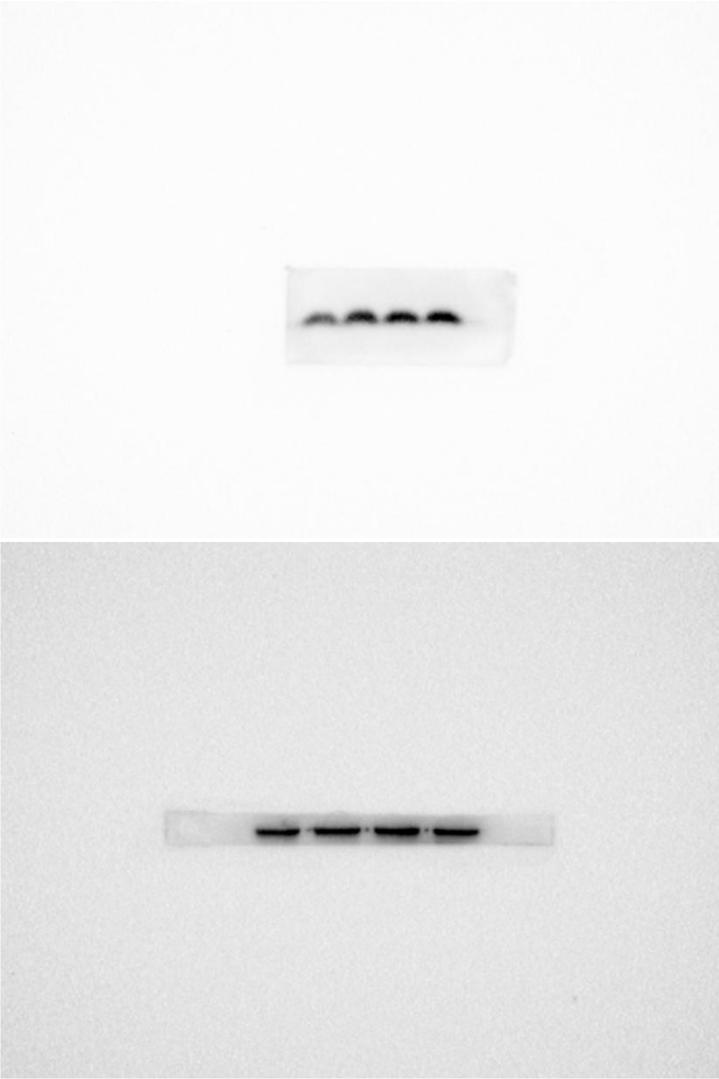

Full-length blots of ESR2, PI3KCG, AKT, P-AKT, NK-κB, GSK3β, P-GSK3β, CDK1, Bcl-2, Bax and β-actin. From right to left: 0 μM, 20 μM, 40 μM, 80 μM. The samples derive from the same experiment.

HCT116(Repeated experiments)

All experimental samples and controls were derived from the same experiment and were treated under the same western blotting conditions.

ESR2

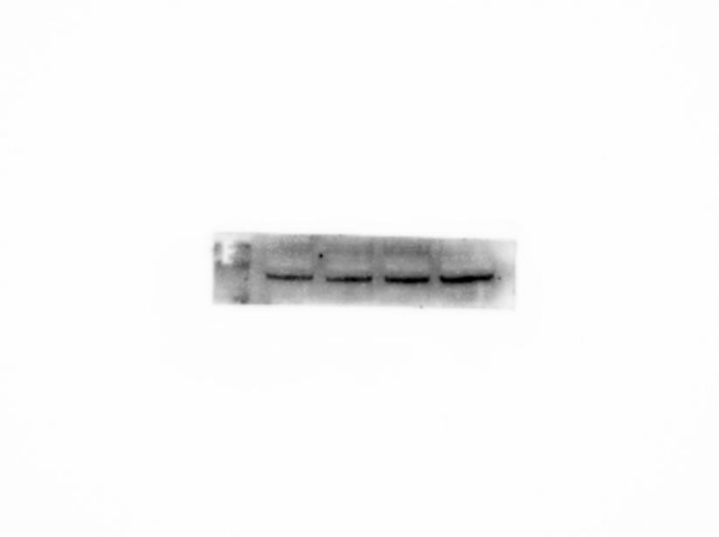

69KD

PIK3CG

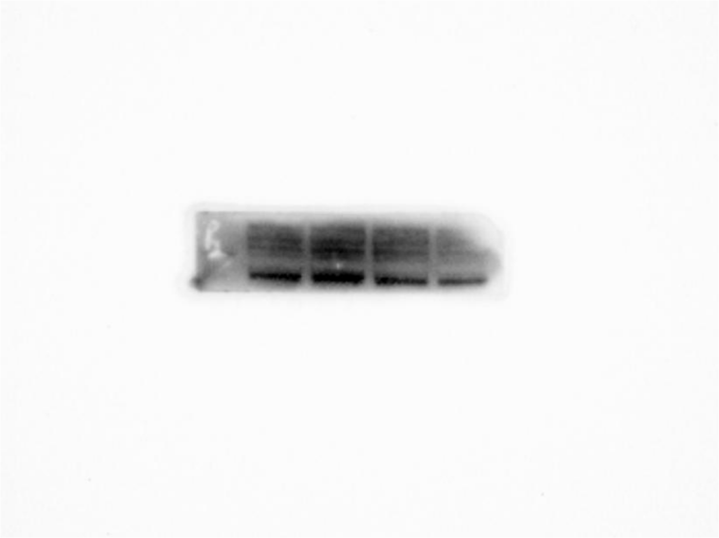

121KD

AKT

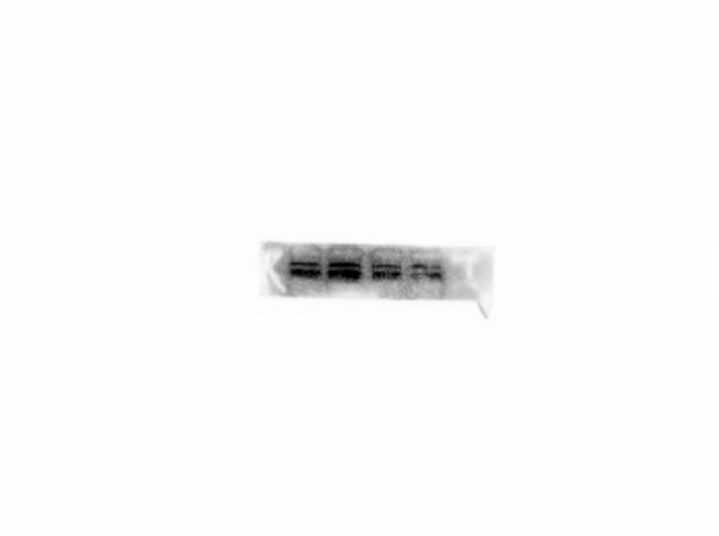

56KD

P-AKT

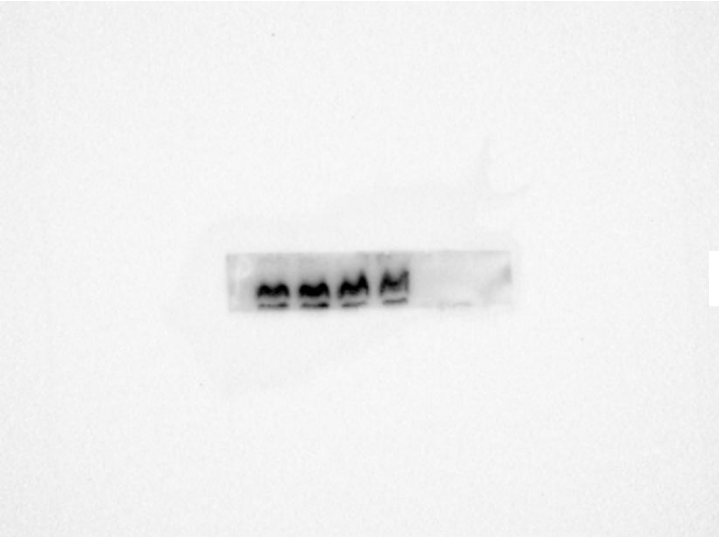

60KD

NF-κB

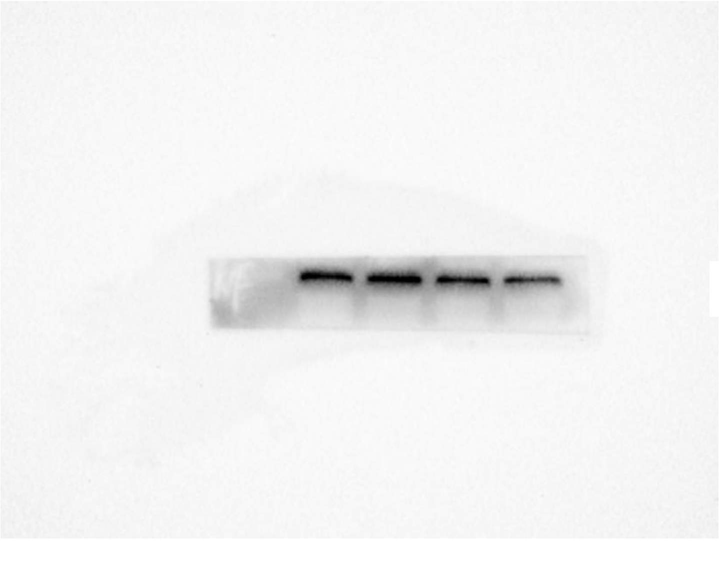

65KD

GSK3β 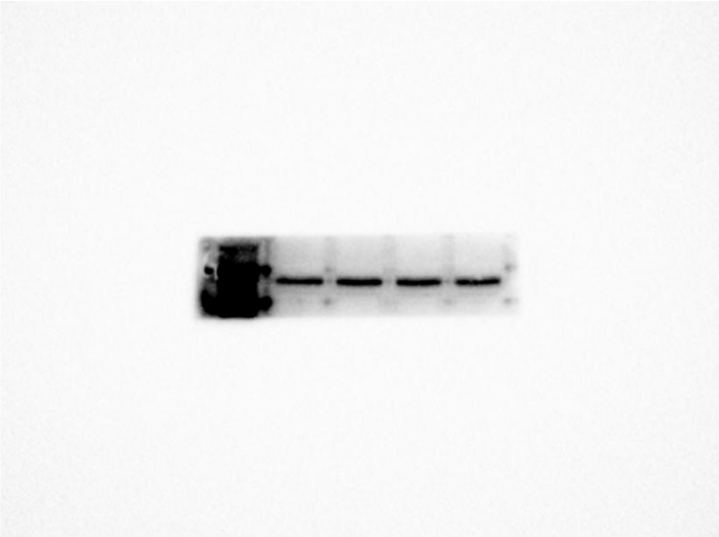 46KD

P-GSK3β 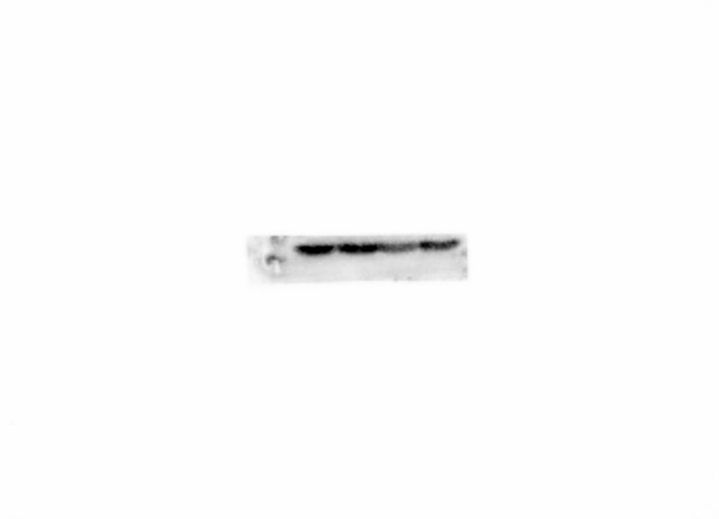 48KD

CDK1 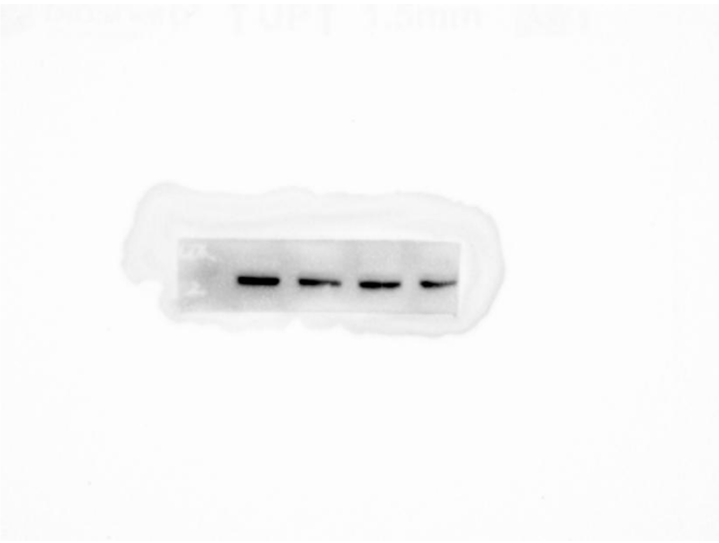 34KD

Bcl2

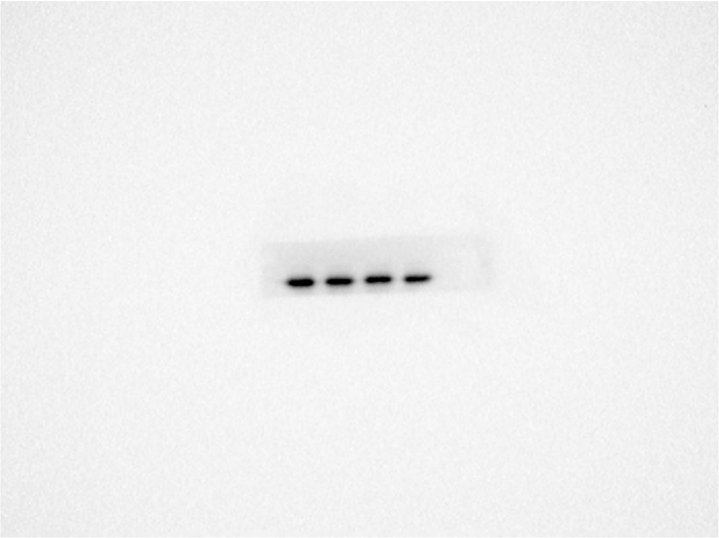

26KD

Bax

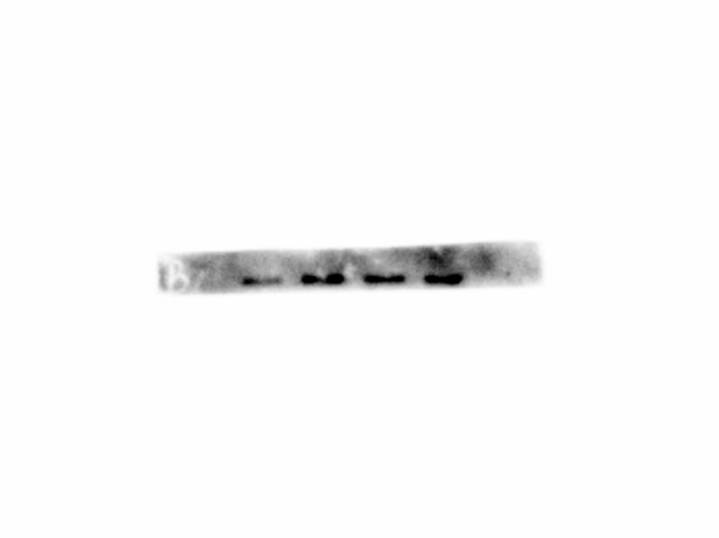

23KD

β-actin

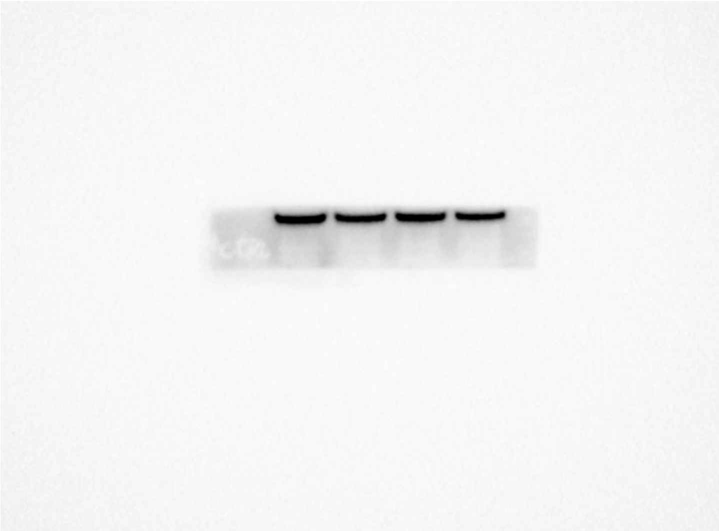

42KD

Full-length blots of ESR2, PI3KCG, AKT, P-AKT, NK-κB, GSK3β, P-GSK3β, CDK1, Bcl-2, Bax and β-actin. From right to left: 0 μM, 20 μM, 40 μM, 80 μM. The samples derive from the same experiment.

SW480(Original images in Fig 5)

All experimental samples and controls were derived from the same experiment and were treated under the same western blotting conditions.

ESR2

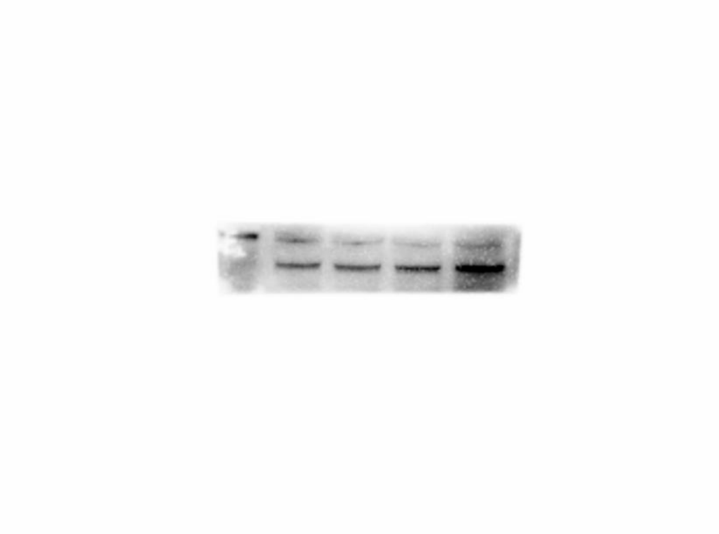

69KD

PIK3CG

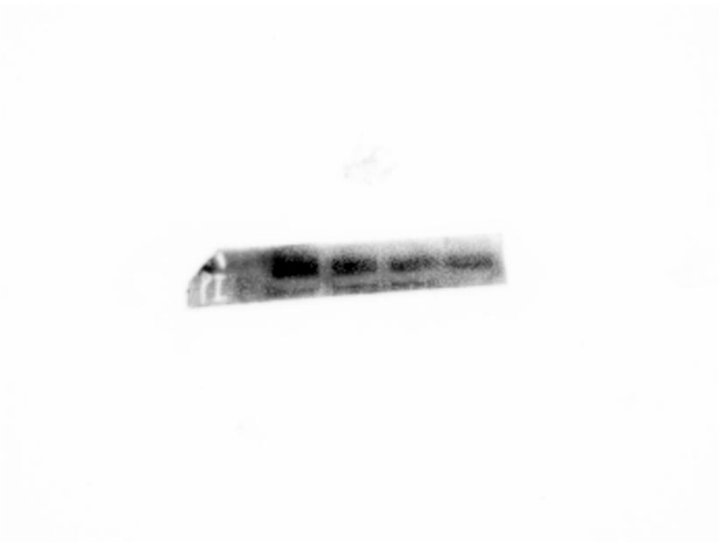

121KD

AKT

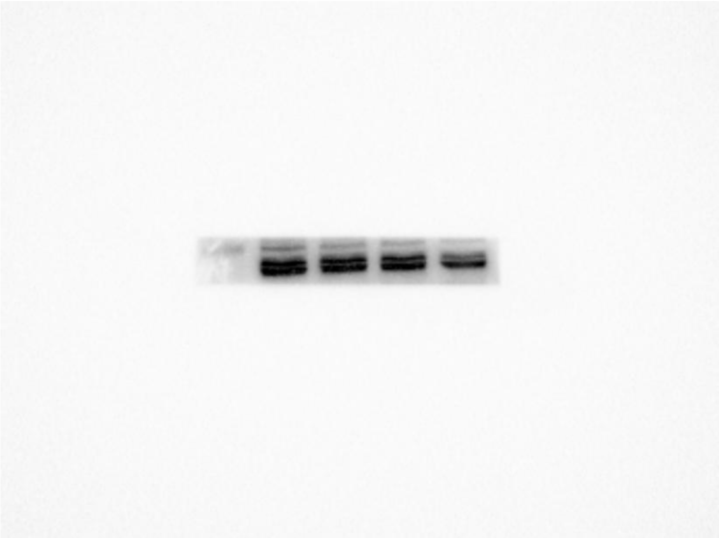

56KD

P-AKT

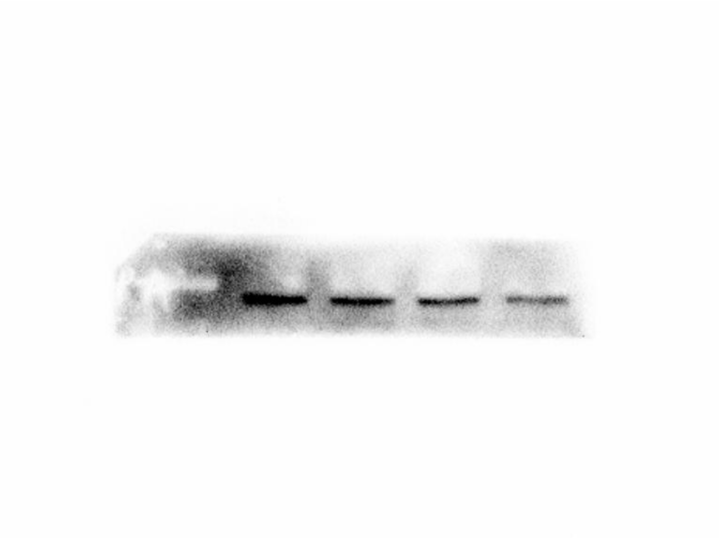

60KD

NF-κB

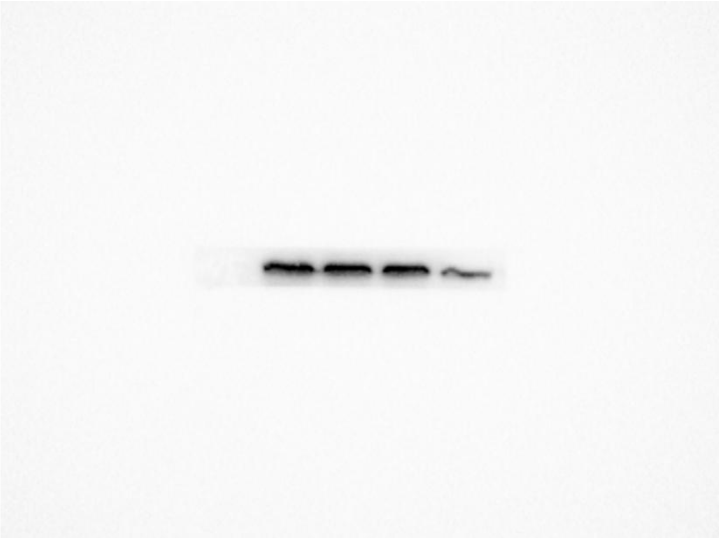

65KD

GSK3β

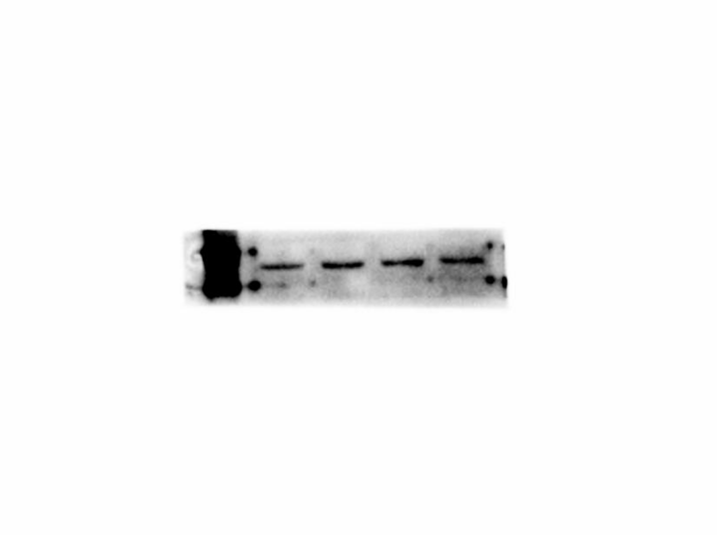

46KD

P-GSK3β

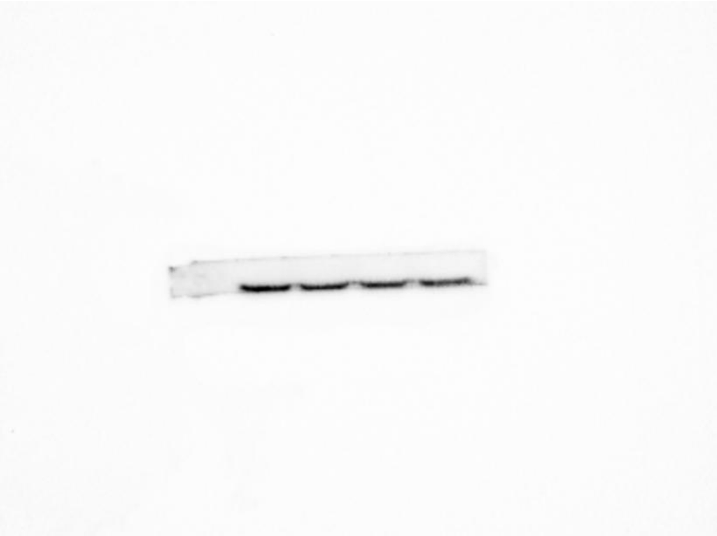

48KD

CDK1

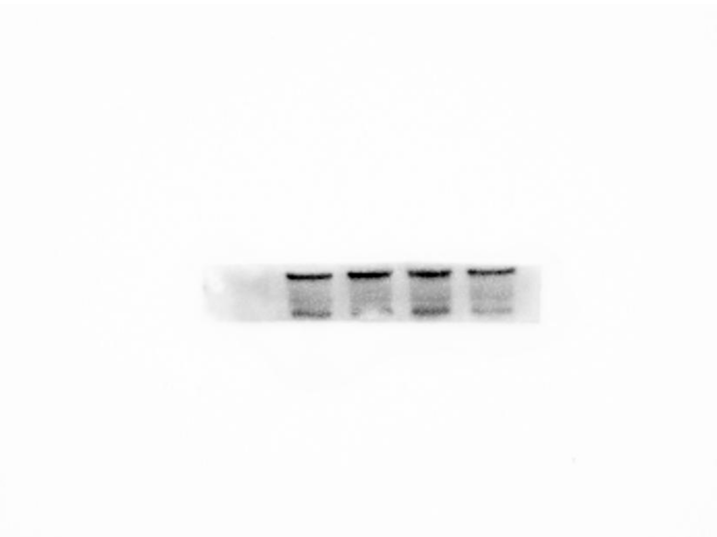

34KD

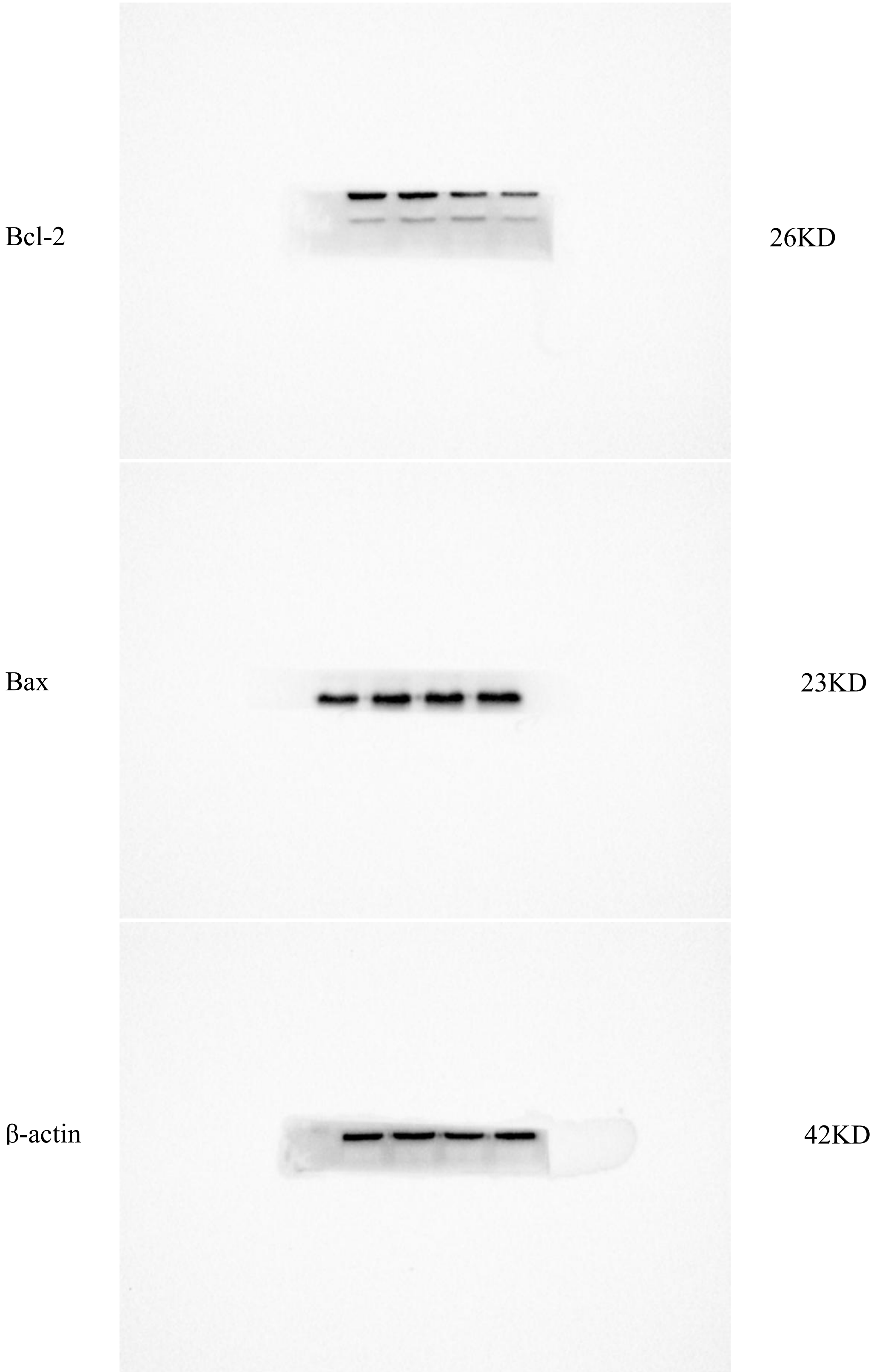

Full-length blots of ESR2, PI3KCG, AKT, P-AKT, NK-κB, GSK3β, P-GSK3β, CDK1, Bcl-2, Bax and β-actin. From right to left: 0 μM, 20 μM, 40 μM, 80 μM. The samples derive from the same experiment.

SW480(Repeated experiments)

All experimental samples and controls were derived from the same experiment and were treated under the same western blotting conditions.

ESR2

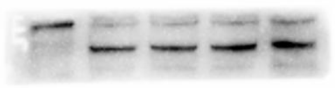

69KD

PIK3CG

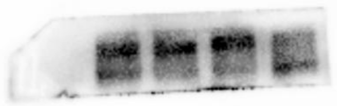

121KD

AKT

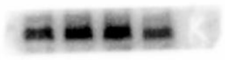

56KD

P-AKT 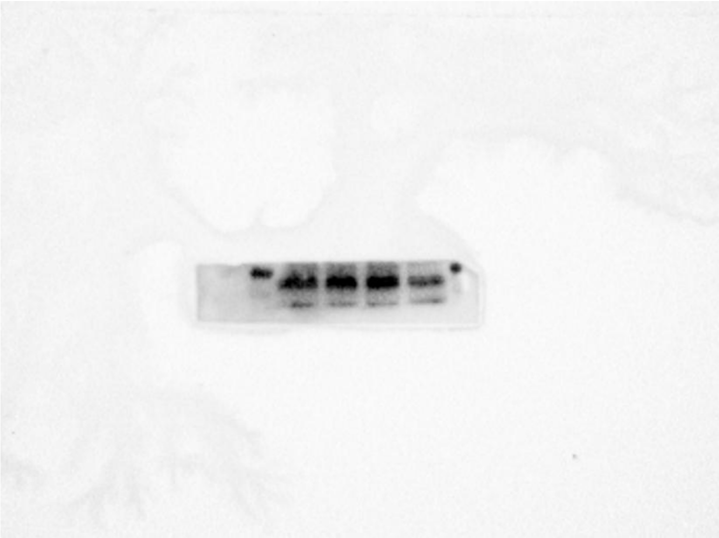 60KD

NF-κB 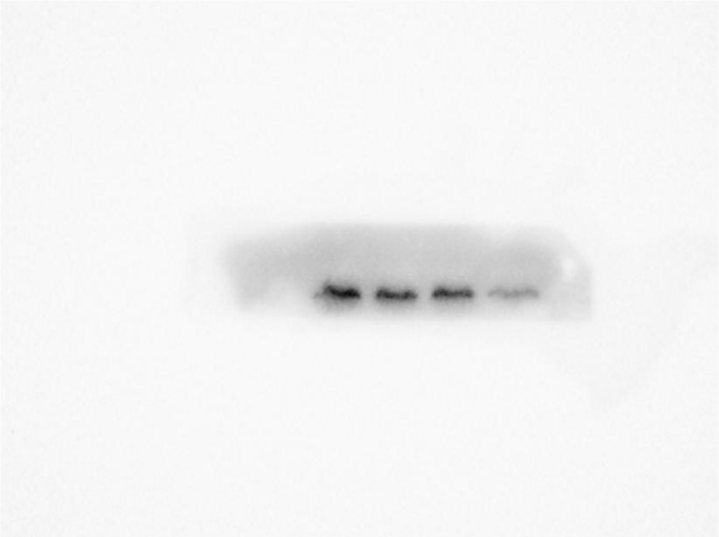 65KD

GSK3β 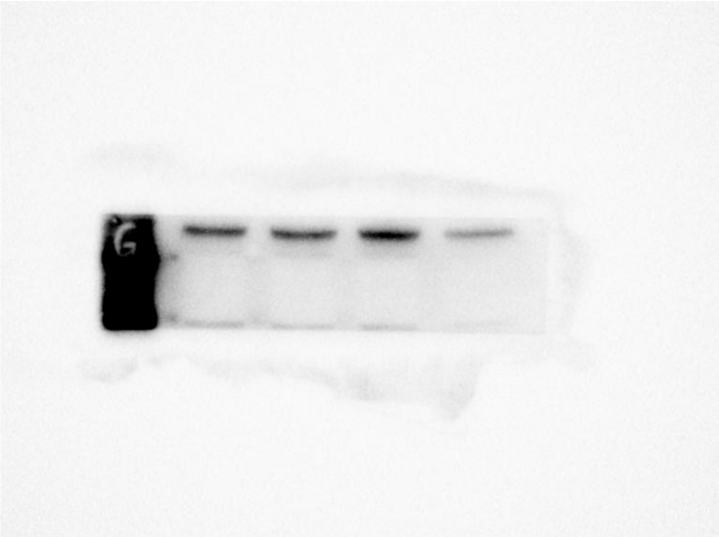 46KD

P-GSK3β

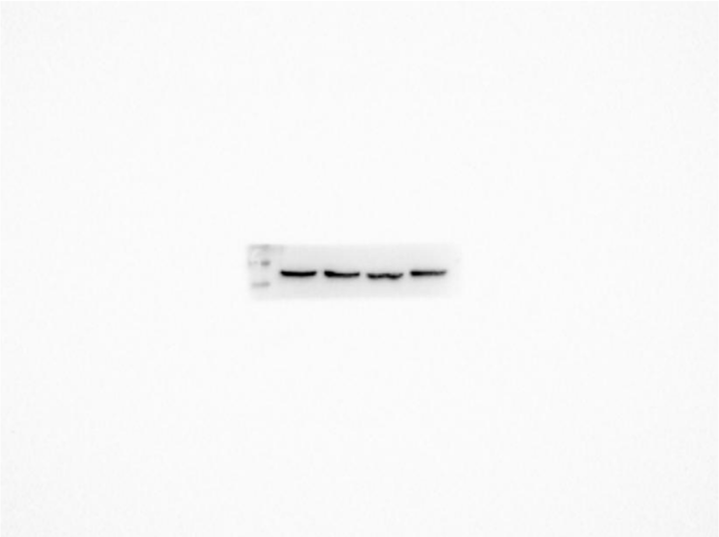

48KD

CDK1

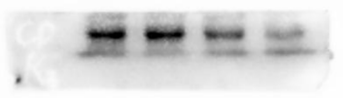

34KD

Bcl2

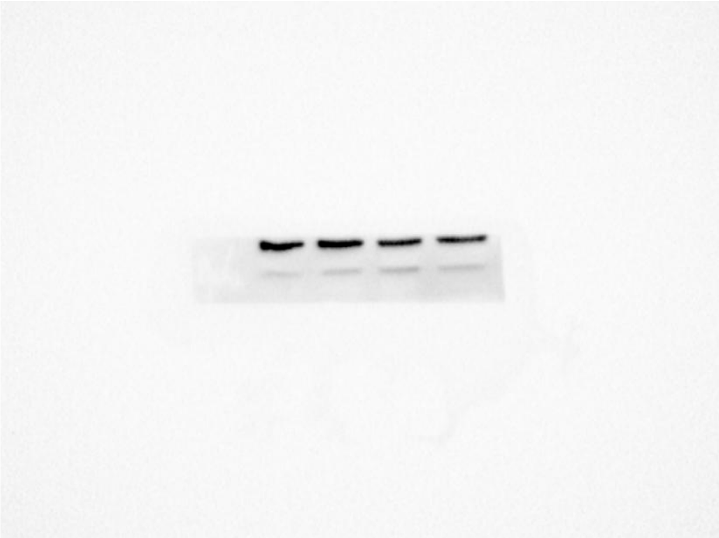

26KD

Bax

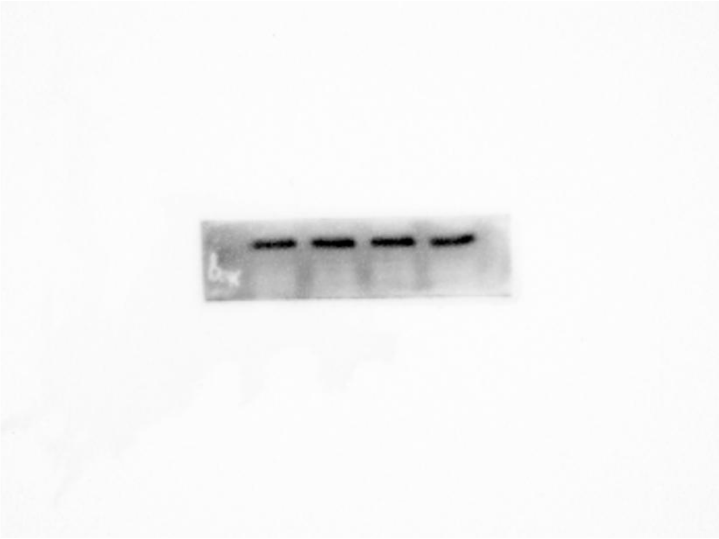

23KD

β-actin

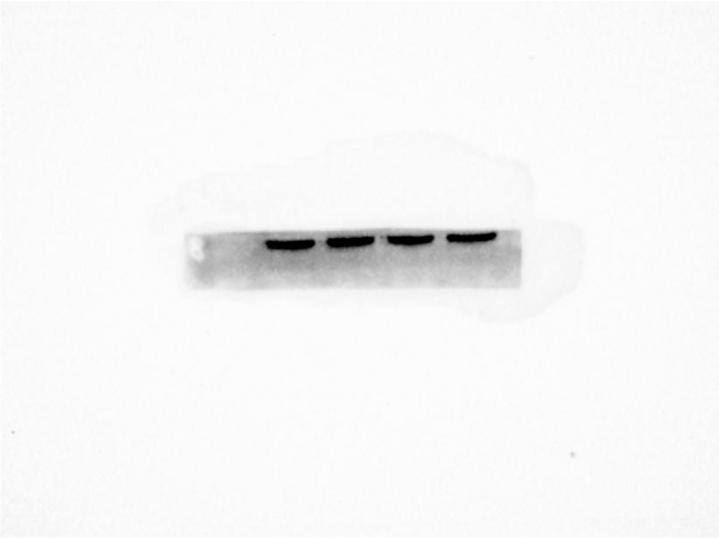

42KD

Full-length blots of ESR2, PI3KCG, AKT, P-AKT, NK-κB, GSK3β, P-GSK3β, CDK1, Bcl-2, Bax and β-actin. From right to left: 0 μM, 20 μM, 40 μM, 80 μM. The samples derive from the same experiment.

SW480(Repeated experiments)

All experimental samples and controls were derived from the same experiment and were treated under the same western blotting conditions.

ESR2

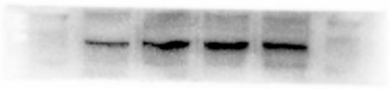

69KD

PIK3CG

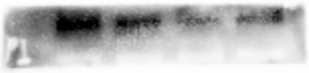

121KD

AKT

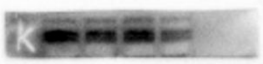

56KD

P-AKT

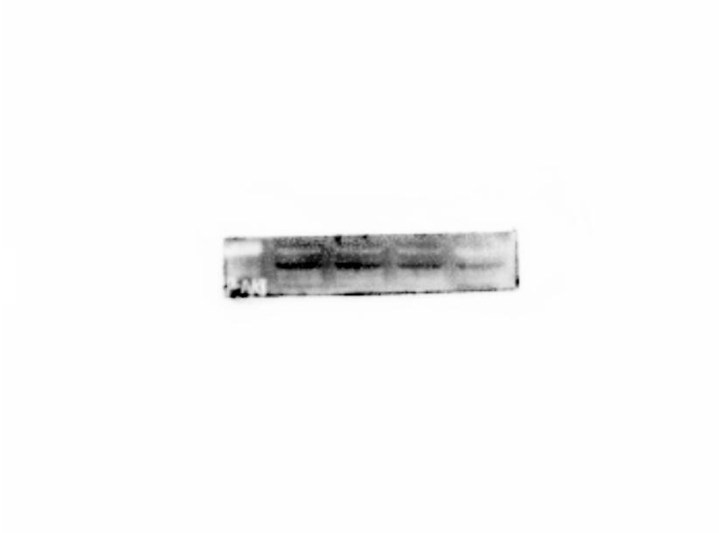

60KD

NF-κB

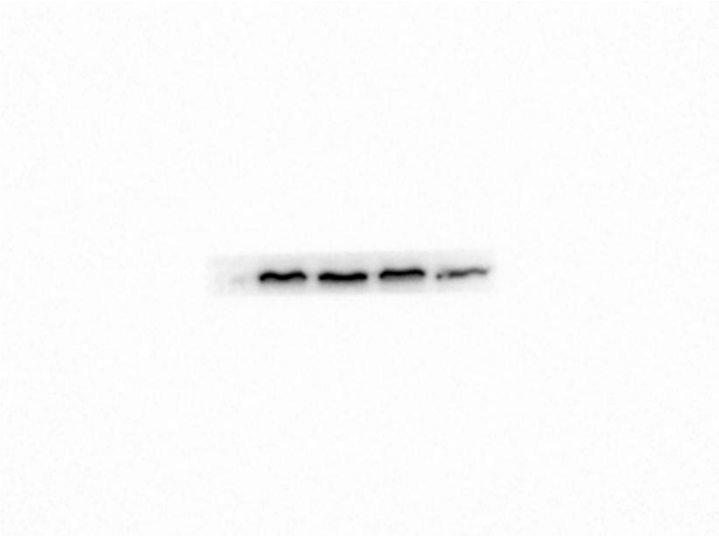

65KD

GSK3β

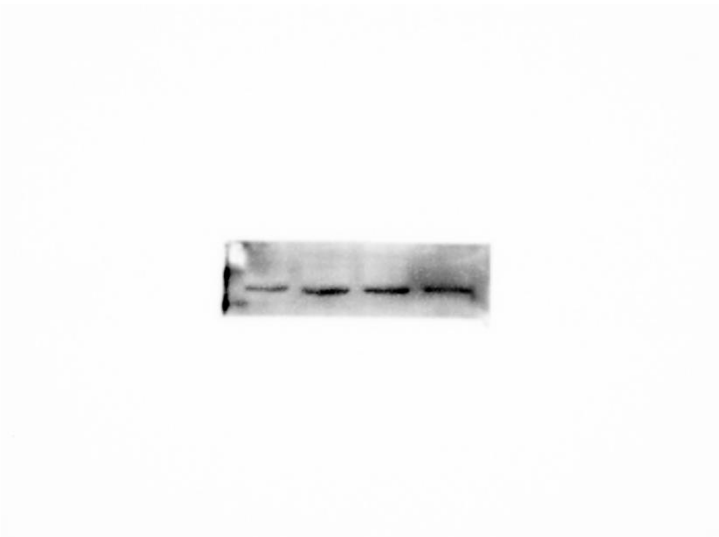

46KD

P-GSK3β

48KD

CDK1

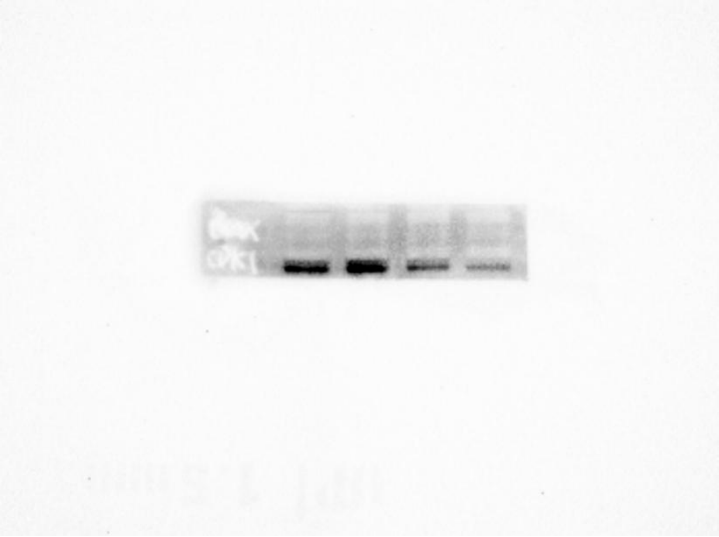

34KD

Bcl2

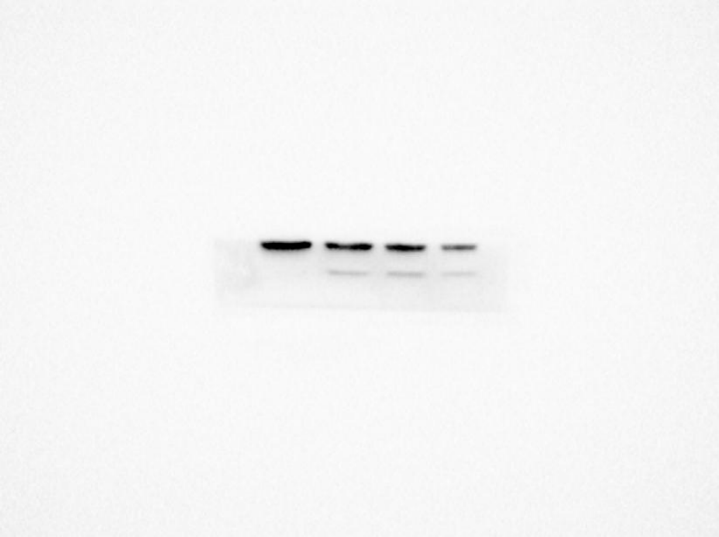

26KD

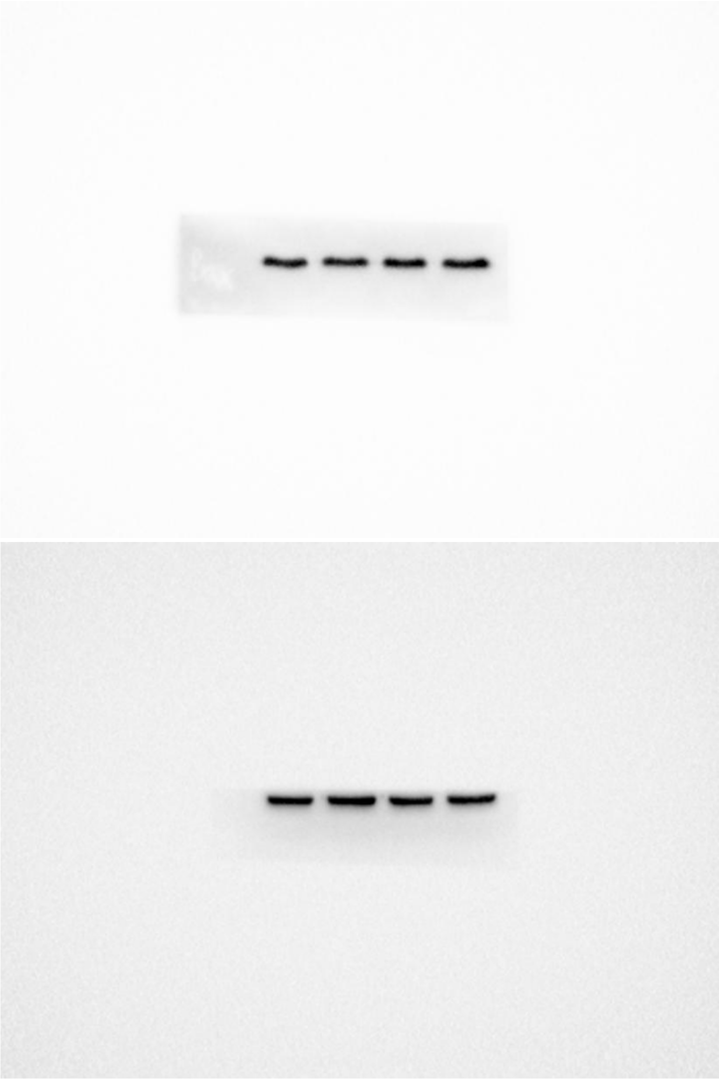

Bax 23KD

β-actin 42KD

Full-length blots of ESR2, PI3KCG, AKT, P-AKT, NK-κB, GSK3β, P-GSK3β, CDK1, Bcl-2, Bax and β-actin. From right to left: 0 μM, 20 μM, 40 μM, 80 μM. The samples derive from the same experiment.
